# Aryl transition metal chemical warheads for protein bioconjugation

**DOI:** 10.1039/c8sc00780b

**Published:** 2018-05-23

**Authors:** Philippe Bisseret, Hajer Abdelkafi, Nicolas Blanchard

**Affiliations:** a Université de Haute-Alsace , Université de Strasbourg , CNRS , LIMA , UMR 7042 , 68000 Mulhouse , France . https://bsm.unistra.fr ; Email: philippe.bisseret@uha.fr ; Email: n.blanchard@unistra.fr

## Abstract

The past seven years have witnessed the burgeoning of protein bioconjugation reactions highlighting aryl transition metal reagents as coupling partners. This new bioorthogonal organometallic chemistry, which sets the scene for stoichiometric processes in place of the catalytic procedures that developed in parallel, already enabled the forging of C–S and C–C bonds onto protein substrates, respectively in their native state or equipped with pre-installed non-natural terminal alkene or alkyne appendages. Although not yet applied to proteins, related transformations pointing to the creation of C–N bonds have, in addition, just been disclosed by targeting peptide lysine residues. Central to this research was the selection of ligands attached to the transition metal, in order to confer to metal complexes, not only their stability in aqueous medium, but also the desired chemoselectivity. We summarize here this body of work, which has already put in the limelight elaborated palladium and gold complexes equipped with biologically relevant appendages, such as fluorescent and affinity tags, as well as drug molecules. This research holds much promise, not only for the study of proteins themselves, but also for the design of new protein-based biotherapeutics, such as protein-drug conjugates or constrained analogs resulting from macrocyclisation reactions.

## Abstract 2

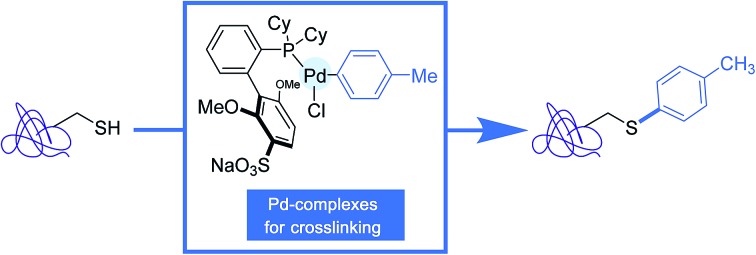
Bioorthogonal organometallic chemistry using aryl transition metal reagents as coupling partners is a burgeoning field that holds great promise notably for the study of proteins.

## I Introduction

Cells teem with proteins which account for about half of their dry mass.^1^ With a limited number of amino acid functionalities, these versatile biomolecules have many functions, ensuring the dramatic acceleration of a myriad of biochemical reactions. In order to comb through their mode of action and dynamics, great effort has been deployed over the past two decades to visualize them in real time, highlighting the use of fluorescent tags, such as the green fluorescent protein isolated from a jellyfish species^2^ or synthetic dyes.^3^ Native fluorescent proteins have been undoubtedly in the limelight as they could be genetically fused to nearly any protein of interest. However, despite its performance, this technique has been hampered by the large size of the fluorescent partner which may impose too important a structural perturbation to the protein under investigation.^4^

In order to address this limitation, much effort has gone into tagging proteins with small synthetic fluorophores, as well as other probes.^3^ For the chemist trained at running reactions on relatively small size molecules in solution in organic solvents, the site-selective functionalization of a protein solubilized in an aqueous medium could already be quite challenging but the situation turns into a daunting task if the desired transformation has to be run in complex biological settings. However, by merging chemistry and protein engineering,^5^ many pitfalls concerning the functionalization of proteins, even in living cells, have been overcome in the past decade. In particular, based on pioneering research from the Schultz group,5*c* the genetic encoding of proteins with amino acids possessing functionalities not encountered in Nature has been rewarding. Thanks to the amber stop codon technique, with minimum perturbation to their function, many proteins can be site-selectively modified with a wide range of appendages, featuring notably *para*-iodophenyl, azide, alkyne or tetrazine functionalities, which are amenable to further derivatization by making use of reactions non-perturbing to biological systems.^5^ In the forefront of these ≪bioorthogonal≫ reactions, as dubbed by Bertozzi,^6^ stands the Cu(i)-catalyzed azide–alkyne cycloaddition (CuAAC) reaction,^3^,^7^ independently reported by the groups of Sharpless and Meldal in 2002.^8^,^9^ Benefiting from many of the attributes that characterize a click reaction, as originally disclosed by Sharpless,^10^ the CuAAC reaction greatly helped to monitor the activity and targets of numerous proteins in the course of *in vitro* studies or experiments at the cell surface. In most of the recent cases, CuAAC bioconjugations performed quite rapidly, with second order rate constants up to 10^2^ mol^–1^ s^–1^, provided copper ions were used at a concentration in the range of 10 μM to 100 μM that, by far, exceeded the one of the protein under scrutiny.3e,^11^ For the organic chemist that routinely uses 1 to 5 mol% of catalyst in transition metal catalyzed experiments, this may appear quite odd. However, the need for Cu(i) in excess in bioconjugation reactions stems from the presence in the protein under study itself, and even more in the biological milieu, of a great number of nucleophiles, and notably amines and thiols, that may rapidly deactivate the catalyst before it plays its role. In spite of its advantages, the CuAAC reaction has been slow to transition within cells, being stymied by cytotoxic effects related to the presence of excess copper(i) species.^12^ In order to remedy this concern, several lines of research were developed, highlighting either non-catalyzed cycloaddition reactions or catalytic procedures displaying a less-toxic transition-metal.

In the case of bioorthogonal cycloadditions, Bertozzi *et al.* reported a non-metal variant of the CuAAC reaction, referred to as the strain-promoted azide–alkyne cycloaddition (SPAAC) reaction, which highlights the utilisation of cyclooctyne scaffolds in place of terminal alkynes.^3^,^13^ In the same vein, alkenes, such as *trans*-cyclooctene or highly strained cyclopropene derivatives, have been used since 2008 as dienophiles in inverse-electron demand Diels–Alder (IEDDA) reactions, featuring tetrazines as electron-poor enophilic partners.^14^ As they do not rely on the utilisation of a toxic metal, both types of strain-promoted cycloadditions have been widely used for *in vivo* studies, although they suffered in some instances from limitations either due to the sensitivity of cyclooctynes to endogenous thiols or, in the case of IEDDA reactions, to the sensitivity to hydrolysis of the tetrazine derivatives. With the fastest bioorthogonal reaction rates reported to date, Diels–Alder ligations have become an unrivalled tool to decipher protein traffickings that occur on the minutes-to-seconds time scale.3e,^14^ On the other hand, the past decade witnessed new ways of tagging proteins with fluorophores or other probes based on palladium-catalyzed reactions that strongly impacted modern organic synthesis, such as the Suzuki–Miyaura, Mizoroki–Heck and Sonogashira cross-coupling reactions.^15^ Initial forays in this line of research were run in the 2005–2011 period and focused on *in vitro* bioconjugation experiments.^16^ Although only low conversions were observed by making use of water-soluble phosphine derivatives as Pd-ligands,16b,c switching to 2-amino- or 2-dimethylamino pyrimidine derivatives was much more rewarding, yielding nearly full conversions in derivatized proteins.16d,e Shortly after, the group of G. Davis realized the first Pd-mediated cross-coupling reactions on an *E. coli* cell surface, targeting a genetically tagged aryliodide-containing protein.^17^ At nearly the same time, the Lin group reported the first Pd-catalyzed chemistry inside bacterial cells, highlighting a genetically encoded alkyne-containing protein substrate.^18^ They provided the incentive from other groups to perform related experiments on proteins located at the surface of mammalian cells as well as on genetically-tagged proteins within *E. coli* cells.^19^ This burgeoning area in palladium chemistry has been the topic of a few recent excellent reviews^15^ and will not be detailed further here. Although quite attractive, the pallado-catalyzed forging of C–C or C–X bonds involving proteins inside living cells as coupling partners remains challenging, being plagued by unproductive interactions of the catalyst with endogenous functional groups, as in the case of copper-catalyzed bioconjugation processes.^20^

In order to address these limitations, efforts are being made at designing transition-metal catalysts of reduced fragility in the biological milieu, including the use of palladium nanoparticles.^21^ In a complementary approach, a small body of research has been recently devoted to the synthesis of isolable, ready-to-use aryl organometallic reagents of limited cellular toxicity, and to their utilization in the derivatization of proteins.^22^–^27^ Our aim in this review is to describe these non-catalytic organometallic cross-coupling reactions. In the first part of the review, we will focus on investigations run in the 2011–2014 period, which highlight the utilisation of aryl palladium reagents in protein bioconjugation reactions by C–C bond formation.^22^–^25^ In the second part, the forging of C–S or C–N bonds onto proteins, by making use of either palladium or related gold reagents, will be examined.^26^,^27^

Insofar as they pave the way for further investigations on proteins, reports of the year highlighting short peptides as substrates will also be briefly discussed in this section.^28^–^30^

## II C–C bond formation

**Scheme 1:**
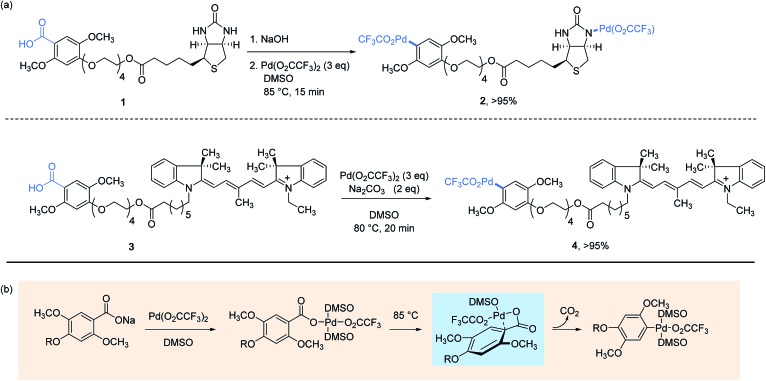
Preparation of aryl palladium(ii) reagents from benzoic acid derivatives (a) and the mechanistic pathway established on sodium 2,4,5-trimethoxybenzoate (R = CH_3_) (b).^32^

The concept of utilizing stoichiometric organopalladium(ii) complexes as reagents for protein bioconjugation was first investigated in 2011 by Myers and co-workers in the course of performing Heck-type cross-coupling reactions involving styryl-modified proteins as the alkene partners.^22^ This groundbreaking work sets the scene to arylpalladium(ii) trifluoroacetates **2** and **4**, respectively conjugated with biotin and indocyanine dye label which displayed a remarkable stability in air as well as in aqueous buffer solutions at ambient temperature. They could alternatively be stored frozen in DMSO at –20 °C over a period of several months without showing signs of degradation. As presented Scheme 1, their preparation in impressive yields points to a decarboxylative palladation reaction in the last step from the corresponding 2,5-dimethoxybenzoic acid derivatives. Taking into account the complexity of the Pd-complexes obtained, as well as their water and air tolerance, this synthesis undoubtedly represents the ice on the cake of a strategy disclosed a decade earlier by the authors for generating arylpalladium(ii) species and which proved to work particularly well with electron-rich sodium benzoates as substrates.^31^

The mechanism that underpins the decarboxylative process is worthy to note here as it has been established, using sodium 2,4,5-trimethoxybenzoate as a model substrate, to proceed by the formation of a distinctive four-membered ring palladacycle, wherein the Pd(ii) center is bonded to the *ipso*-carbon atom of the arene ring.^32^

**Scheme 2:**
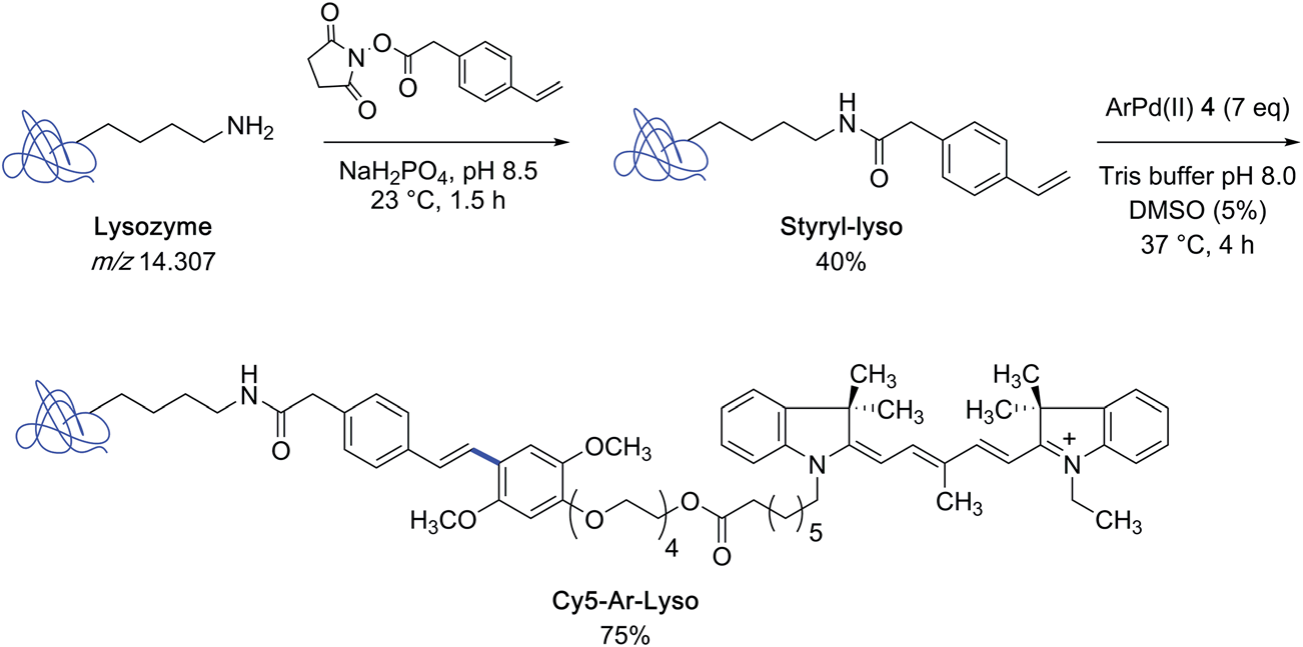
Preparation of styryl-modified lysozyme and synthesis of an indocyanine dye lysozyme bioconjugate.

With the indocyanine-bearing aryl palladium(ii) complex **4** on hand, the authors explored the feasibility of labeling a protein substrate that had been covalently modified to present a styryl appendage. For that purpose, they selected the archetypical lysozyme *m*/*z* 14 307 as the protein partner and converted it into a mixture of styryl derivatives, comprising the singly modified lysozyme as major constituent, by using styryl succinimide as the acylating agent. After incubation of the latter mixture in Tris buffer containing 5% DMSO at 37 °C in the presence of an excess of reagent **4**, the singly-modified Heck-type coupling product could be isolated in 75% yield (Scheme 2).

**Scheme 3:**
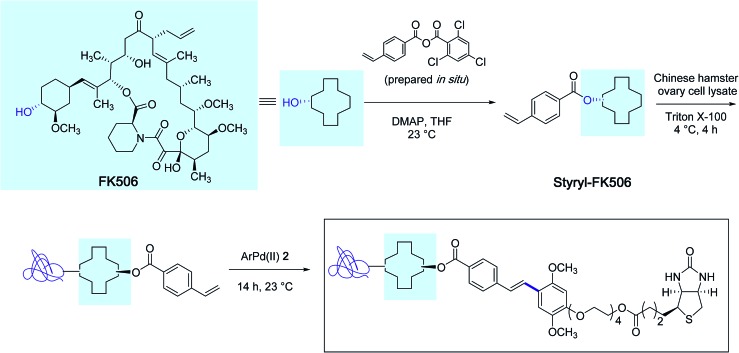
Affinity-enrichment experiment with macrolide FK506 and Chinese hamster ovary cell lysate.

With the biotin-conjugated arylpalladium(ii) reagent **2**, the authors further demonstrated the potential of their method in the course of an affinity-enrichment experiment featuring styryl-modified FK-506 macrolide lactone as the probe (Scheme 3). The protein solution obtained from Chinese hamster ovary cell lysate was first treated with styryl-FK506 during 4 h at 4 °C and incubated further with the biotinyl reagent **2** near room temperature during 14 h to deliver, after purification, proteins complexed to the biotin-conjugated probe molecule.

**Scheme 4:**
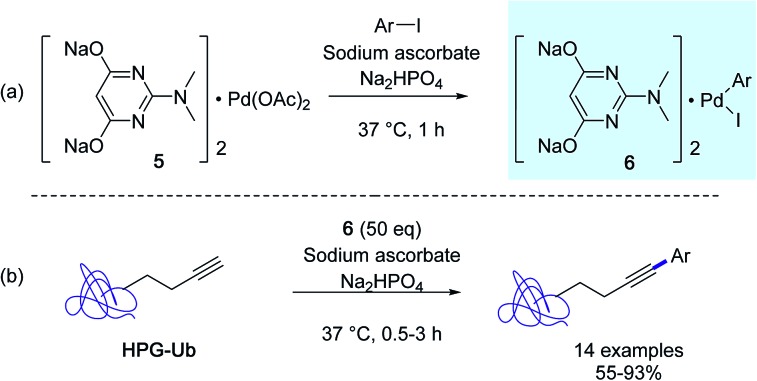
(a) Sonogashira cross-coupling reactions with HPG-Ub, (b) preincubation step adapted from ref. 33.

At about the same time, the Lin group developed a new protein bioconjugation strategy highlighting copper-free Sonogashira cross-couplings in aqueous medium with homopropargylglycine-encoded ubiquitin (HPG-Ub) as the protein substrate and aminopyrimidine–palladium complex **5** as the catalyst (Scheme 4).^33^ In order to achieve high conversions in arylated HPG-Ub, they employed a two-step protocol in which the oxidative addition complex **6** was generated first followed by the arylation reaction with the protein substrate. It was suggested that the pre-incubation step was required in order to alleviate nonspecific sequestration of the palladium complex **5** by the proteins. However, for optimal conversions, a large excess of *in situ* generated arylpalladium(ii) complex was required, because of its tendancy to decompose gradually in buffer solution.

**Scheme 5:**
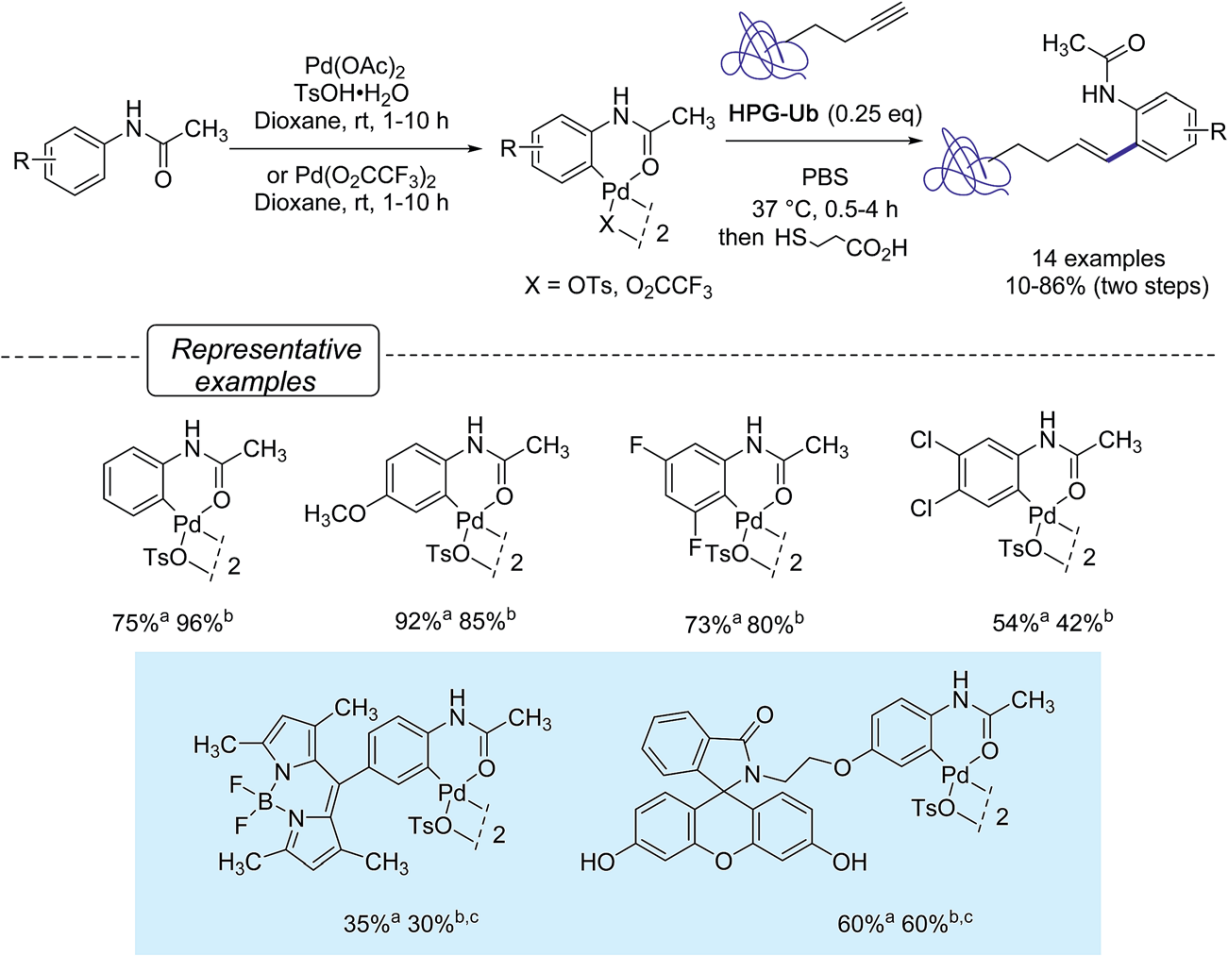
Synthesis of palladacycles from acetanilides and reactions with HPG-Ub. ^a^Yield of formation of palladacycle; ^b^Yield in bioconjugated protein; ^c^25 eq. of palladacycle were used.

Aiming at further developing their bioorthogonal C–C bond forging chemistry on proteins, Lin and co-workers^23^ were inspired by Myers' work and focus their efforts at elaborating more robust arylpalladium(ii) complexes that could be fully characterized and stored as ‘ready-to-use’ reagents. They ultimately discovered that a range of palladacycles, easily available from acetanilide derivatives as previously reported on acetanilide itself,^34^ exhibited a good stability in aqueous buffer and were capable of arylating HPG-Ub with moderate to high yields in phosphate buffer solution (PBS) at 37 °C, when used in excess of 4 equivalents. Under these conditions, as shown in Scheme 5, styryl derivatives were obtained in place of aryl alkynes resulting from Sonogashira cross-coupling reactions. This was certainly unexpected as closely related palladacycles had already been precognized for copper-free Sonogashira cross-coupling procedures.^35^

**Scheme 6:**
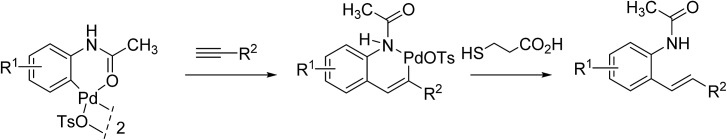
Proposed mechanism for the generation of styrene products.

Quite remarkably, the bioconjugation protocol could perform, not only with palladacycles derived from simply substituted phenyl rings, but also with more elaborate derivatives adorned with a fluorescein- or a BODIPY dye label, although rather sluggishly in the latter cases. The postulated mechanism for the formation of styrenes highlights, as outlined in Scheme 6, a carbopalladation step resulting in the formation of a vinylpalladium(ii) intermediate that undergoes a reductive depalladation step upon quenching of the reaction mixture with 3-mercaptopropanoic acid.

**Scheme 7:**
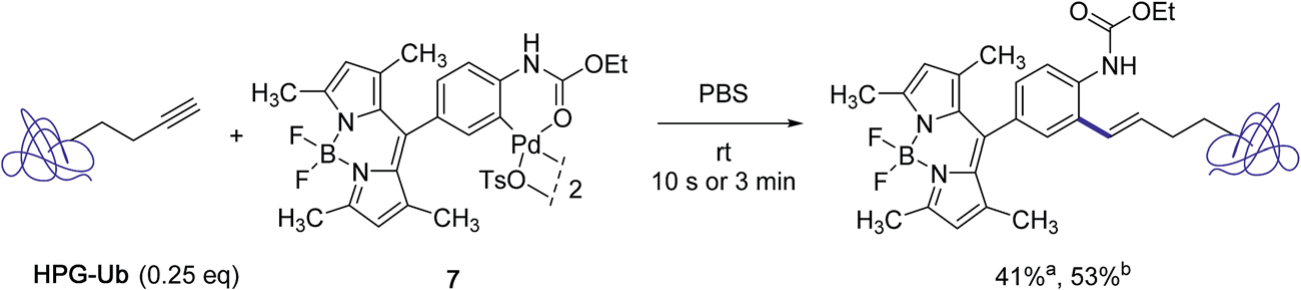
Reactivity of the BODIPY-substituted ethyl-*N*-phenylcarbamate palladacycle **7** toward HPG-Ub. ^a^Conversion after 10 s; ^b^conversion after 3 min.

The subsequent year, the Lin group disclosed a significant improvement to their Heck-like bioorthogonal cross-coupling procedure that showcased the utilization of *N*-phenylcarbamate palladacycles in place of acetanilide derivatives.^24^ While keeping an excellent stability in buffer solution at room temperature for 24 hours, the new derivatives exhibited quite impressive reactivities in the bioconjugation reaction with the alkyne-encoded protein HPG-Ub, affording in nearly all cases the desired styrene-derived protein in quantitative conversion after 3 min. In the particular case of the BODIPY-derived palladacycle **7**, although full conversion could not be reached, a correct yield of the tagged protein could be obtained already after a brief treatment of 10 s (Scheme 7). The new protocol afforded a clear improvement to the utilization of the acetanilide BODIPY equivalent where a conversion of 30% only was observed after 30 min with an excess of 25 equivalents of the palladacycle reagent (Scheme 5).

Before closing this section on the use of arylpalladium(ii) reagent in protein bioconjugation reactions by C–C bond formation, it is worthy to note that both methods described so far yield to Heck-like cross-coupling derivatives and may be used to tag proteins with a fluorophore and/or a biotinyl moiety. Although up to now restricted to *in vitro* protein derivatization, they are opened to experiments under cellular settings as they put in the forefront styryl and alkynyl-modified proteins which are not encountered in Nature.

With impressive reaction kinetics and the utilization of a co-solvent-free buffer medium, Lin's latest protocol favorably competes with most of the reported bioconjugation chemistries based on alkynyl protein substrates.^3^,^11^ Myers' study remains however a prime source of inspiration that should deserve a particular attention in instances where primary alkynes could not be used due to their lack of stability and notably their propensity to dimerize.15f,g In the case of both peptide or protein substrates, arylation reactions performed remarkably with complete chemoselectivity, targeting only the pre-installed unsaturated handles and leaving untouched amino and hydroxy functionalities notably from lysine, serine and threonine residues. This new bioorthogonal C–C forging chemistry would benefit from the examination of a broader substrate scope, including in particular cysteine-containing proteins. These seminal experiments proved to performed quite efficiently with 10 times less Pd-equivalents than the majority of the catalytic variants reported so far.^15^–^18^ Along this line, in the context of *in vivo* experiments, these bioconjugation protocols should not be significantly hampered by cytotoxic issues associated to the use of palladium species.^36^

## III C–S bond formation

Following a similar concept of introducing aryl moieties in proteins with the aid of aryl transition metal reagents, the Wong group tackled the possibility of forging C–S bonds by derivatizing the sulfhydryl group in cysteines.^25^ Their aim was notably to offer a robust alternative to the *N*-methylmaleimide cysteine ligation which, although commonly used, has been thwarted by limitations due to the lack of stability in physiological environments of the maleimide adducts.^3^,^37^ For that purpose, in place of palladium-based reagents, they put in the forefront their savoir-faire in organogold chemistry, highlighting the great potential of 6-membered ring cyclometallated gold(iii) reagents such as the complex **8** equipped with a *N*,*N*′-bis(methanesulfonyl)ethylene diamine ancillary ligand.^38^

**Scheme 8:**
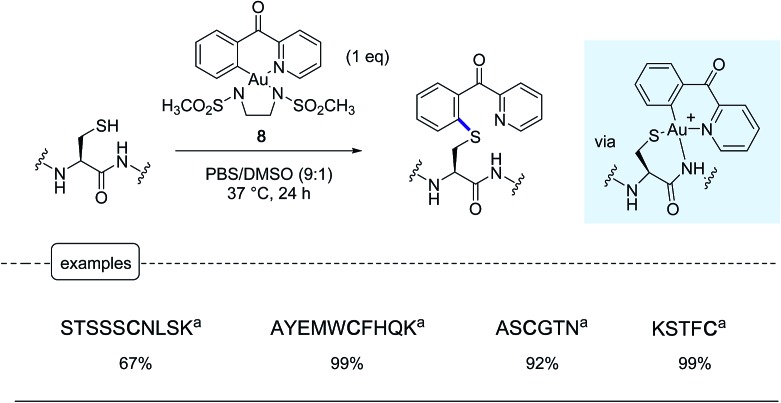
Modification of peptides by the cyclometalated gold(iii) complex **8**. ^a^Peptide sequence.

In proof-of-concept experiments performed on a palette of short peptide substrates, remarkable conversions in corresponding aryl thioethers could be obtained by exposure at 37 °C for 24 h to an equimolar amount of gold complex **8** (Scheme 8). Under these conditions, the reaction did not stop at the gold peptide adducts shown in the Scheme but directly furnished the arylated product resulting from a reductive elimination step rarely encountered in organogold(iii) chemistry.

**Scheme 9:**
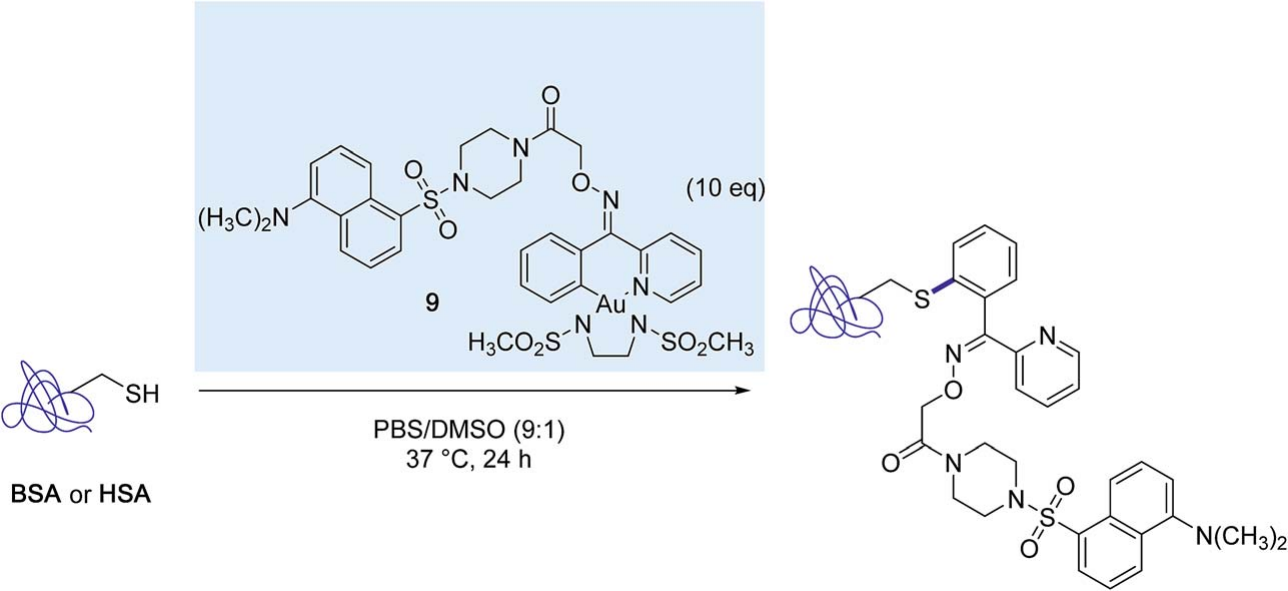
Modification of bovine serum albumine (BSA) and human serum albumin (HSA) mediated by the dansyl-linked gold(iii) complex **9**.

The excellent chemoselectivity of the reaction was further demonstrated in bioconjugation reactions targeting the surface exposed cysteine residue of bovine serum albumin (BSA) and human serum albumin (HSA), with the aid of the distinctive dansyl-linked gold(iii) complex **9** (Scheme 9).

**Scheme 10:**
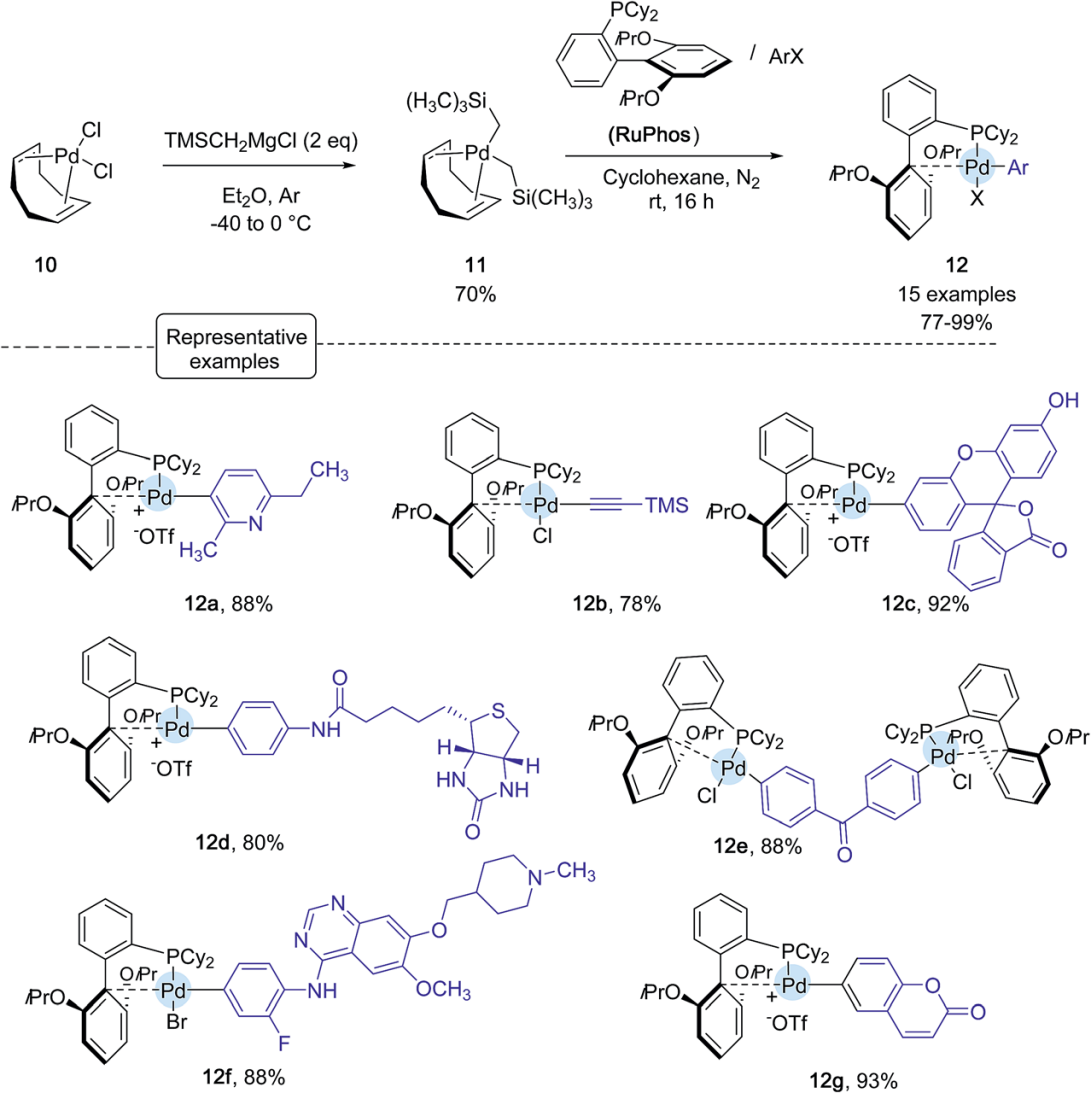
Preparation of arylpalladium(ii) complexes.

Shortly after Wong's work, the Buchwald group reported another stunning method to affix bioorthogonal motifs to cysteine residues involving, this time, aryl palladium(ii) reagents.^26^ Taking into account that Pd and thiols had previously been considered incompatible for palladium-catalyzed cross-coupling reactions, this contribution acutely emphasized the group's expertise in the chemistry of Pd complexes. As shown in Scheme 10, a range of arylpalladium(ii) complexes were in the first place prepared from (COD)PdCl_2_**10** in line with a protocole independently established by the Buchwald and Beller groups highlighting the use of the bis-silyl palladium derivative **11** as the source of Pd(0).^39^ In a nitrogen-filled glovebox, oxidative addition complexes were obtained in good to excellent yields. They displayed a vast scope of biologically relevant groups encompassing fluorescent tags (**12c**, **12g**), bioconjugation handles (**12b**), an affinity tag (**12d**) as well as a drug molecule (**12f**). Quite remarkably, in spite of their elaborated structures, these palladium(ii) complexes exhibited an excellent stability under ambient conditions, and could be stored for several months in closed vials under air at 4 °C.

**Scheme 11:**
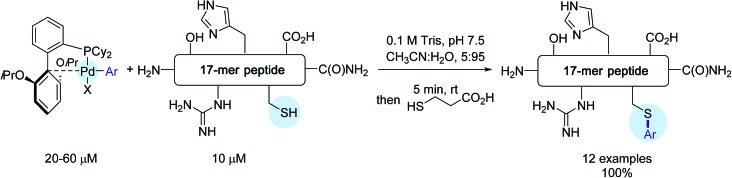
Arylation of the 17-mer peptide H_2_N-RSNFYLGCAGLAHDKAT-C(O)NH_2_.

The reactivity of these complexes was first showcased by selecting a cysteine-containing 17-mer peptide as the model substrate. By making use of CH_3_CN : H_2_O, 5 : 95 as the solvent as well as Pd-reagents in excess (2–6 eq.), impressive results were obtained. In all cases, the desired S-arylated derivatives were produced in quantitative yield after only 5 min of reaction time (Scheme 11)!

Subsequently, on a model 15-mer peptide containing this time two cysteine residues, the authors tackled the possibility of preparing a constrained analogue by making use of the bis-palladium reagent **12e**. Their aim was to offer an alternative protocol for peptide macrocyclisation reactions that have been quite in vogue recently for designing new biotherapeutics.^40^

**Scheme 12:**
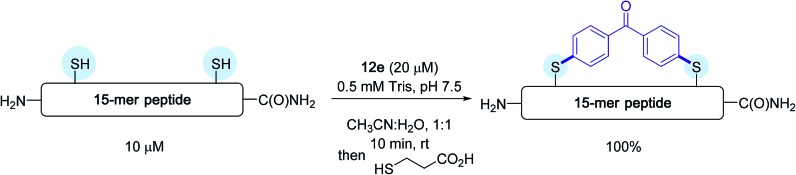
Macrocyclisation reaction of the 15-mer peptide H_2_N-IKFTNCGLLCYESKR-C(O)NH_2_ possessing two cysteines (C) at *i* and *i* + 4 positions.

As indicated in Scheme 12, running the reaction in a 1 : 1 water/acetonitrile solvent mixture in the presence of a twofold excess of bis-palladium complex resulted in quantitative peptide stapling after only 10 min.

**Scheme 13:**
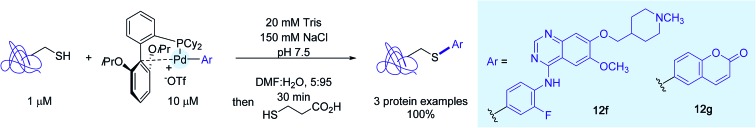
Protein modification using the Vandetanib- and -coumarin-linked palladium complexes **12f** and **12g**.

Bolstered by these results with peptides, the authors turned their attention on proteins. They selected for that purpose three cysteine-containing candidates and, as Pd-containing coupling partners, complexes **12f** and **12g**, respectively associated to the Vandetanib residue and a coumarin dye. In the presence of a tenfold excess of Pd-reagents, cysteines were quantitatively modified with the aryl tag for all three proteins (Scheme 13).

**Scheme 14:**
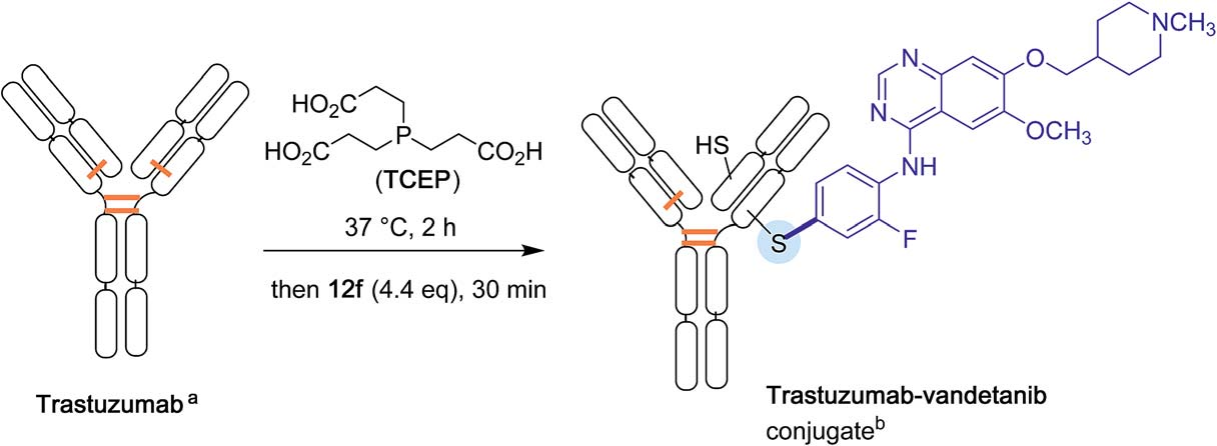
Antibody-drug conjugate formation. ^a^Disulfide bridges are shown in orange. ^b^ Based on the reductive cleavage of only one disulfide bridge.

Again with proteins, the Buchwald group finally explored the possibility of attaching directly drug molecules to cysteine residues in antibodies. Their aim was to forge for the first time antibody-drug conjugates (ADC) in the absence of any link between the protein and the chemical drug.^41^ As shown Scheme 14, partially reduced Trastuzumab antibody, was treated with an excess of the oxidative addition complex **12f** derived from Vandetanib, a much in use brominated anti-cancer drug.^42^ After half an hour of reaction time, an excellent conversion in the expected ADC derivative was found.

Buchwald's method is quite outstanding as, in all cases, a complete chemoselectivity for cysteine among other nucleophilic residues was observed leading, within brief reaction times, to a vast scope of peptide and/or protein S-arylated bioconjugates.

Although most of the palladium-catalyzed C–S bond forming chemistry has relied so far on the utilization of strongly chelating ferrocenyl-based diphosphine ligands in order to minimize unproductive interactions of Pd-catalysts with thiolate nucleophiles,^43^ Buchwald's first palladium-mediated bioconjugation procedure sets the scene to a ligand from the biaryl phosphine family which had been in the limelight in the course of palladium-catalyzed amination reactions performed in the presence of a base.^44^ Such ligands have been shown to possess additional Pd-arene interactions which impart them with a partial bidentate character and tune the electrophilicity of the Pd-center.^45^ Here, in the absence of an external base, RuPhos proves to be sensitive only to the soft nucleophile cysteine thiol.^46^

In spite of its performance, the method still suffers from two main shortcomings. A first one comes from the requirement of a glovebox for the preparation of the oxidative addition Pd-complexes. The second limitation arises from the necessity to run arylation reactions in an aqueous medium that contains an organic co-solvent such as DMSO or CH_3_CN in order to dissolve the palladium reagents.

**Scheme 15:**
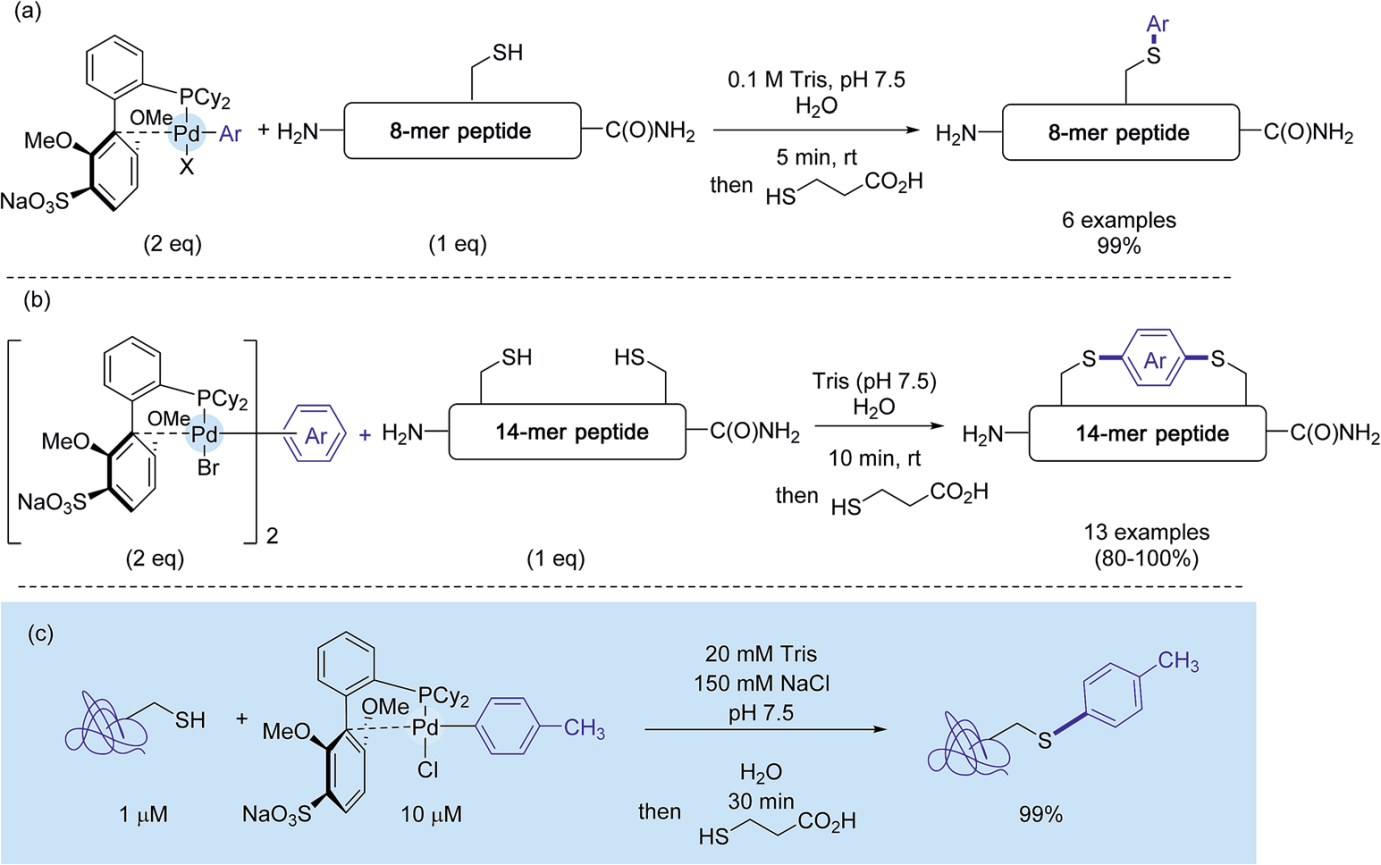
S-Arylation experiments with sSPhos-ligated palladium complexes (a) on the 8-mer peptide H_2_N-TDEYCKSR-C(O)NH_2_, (b) macrocyclisation reactions on the 14-mer peptide H_2_N-ACYKRSDFTCGGGS-C(O)NH_2_, (c) on a model protein.

Concerned by these limitations, the authors eventually disclosed a few months ago an improved version of their method that puts in the forefront the utilisation of sSphos (Scheme 15) as the Pd-ligand.^27^ This biarylphosphine ligand, structurally related to RuPhos, had been precognized ten years before for Suzuki–Miyaura cross-couplings in water.^47^ Here, it plays another key role, enabling the preparation of water soluble Pd(ii) reagents in the open air. Under cosolvent-free conditions, as presented in Scheme 15, these complexes mediated the rapid tagging in nearly quantitative yields, not only of a small peptide but also of a model protein. On peptides again, the authors synthesized a palette of bis-palladium complexes that were used for macrocyclisation experiments of a 14-mer peptide featuring two cysteine residues at position *i* and *i* + 8 (Scheme 15b). Here also, in contrast with the stapling experiments run with the RuPhos-supported bis-Pd reagent **12e**,^26^,^29^ the presence of a co-solvent was not usually required, in spite of the enhanced lipophilicity of stapled peptide analogs compared to the starting peptide substrate.

Let us briefly compare at this stage Wong's seminal procedure with Buchwald's two methods. Although based on the chemistry of two different transition metals, these three protocols have common points: they were realized with a tenfold excess of aryl metal complexes and delivered the desired aryl products with an excellent or complete chemoselectivity. Buchwald's revised method appears definitely the most appealing as it obviates the use of an organic co-solvent, while maintaining very good reaction kinetics. As they were performed on proteins containing either a single cysteine residue or a single exposed cysteine, this new C–S-forging chemistry should be tested further on a wider scope of substrates, comprising in particular several reactive cysteines.

To close this section, one of the latest contribution of the Buchwald group ought to be mentioned. Although it does not concern proteins so far, it sets the basis of a new approach for attaching radiochemical entities onto cysteine residues *via* two palladium-mediated sequential cross-coupling reactions.^30^

**Scheme 16:**
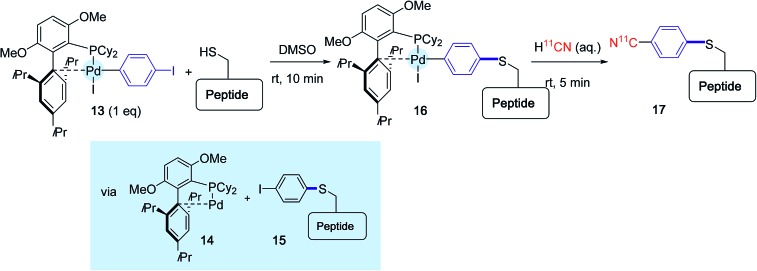
^11^C-Cyanation of a model peptide mediated by the BrettPhos-ligated palladium complex **13**.

The principle of this method is outlined Scheme 16, in the context of ^11^CN-labeling experiments of a model unprotected peptide. The peptide substrate that contained one cysteine residue was submitted first to a brief exposure in DMSO to the oxidative addition complex **13** derived from 1,4-diodobenzene and supported by BrettPhos. This ligand was selected in place of RuPhos or sSPhos because it had been previously precognized for cyanation reactions.^48^ By contrast with the preceding investigations, this first treatment did not principally deliver the *para*-iodophenyl peptide derivative **15**, accompanied by the Pd(0) complex **14**, but led in place to the combination between both species **16** resulting from a subsequent oxidative addition. The reaction medium was further exposed for 5 min to an aqueous solution of radiolabeled H^11^CN, yielding to the desired labeled peptide product **17**.

It is worth mentioning that the duration of the overall process, including HPLC purification at the end, did not exceed 15 min and thus nicely matched ^11^C half life (*t*1/2 = 20 min). In its current format, the method remains plagued by the use of DMSO as the main solvent, which precludes its utilization for protein labeling experiments.^49^

## IV C–N bond formation

The formation of C–N bonds has been a common feature of many bioorthogonal derivatization procedures applied to proteins equipped with a functional handle such as an azide, as exemplified by the gold standard Staudinger ligation and the CuAAC reaction.^3^ Although less frequently, efforts have also been made to modify proteins in their native state *via* C–N bond forging strategies onto lysine residues or N-terminal amino groups, pointing to the utilization of highly electrophilic partners such as ketene derivatives, activated esters or arene diazonium salts.^50^ In spite of their utility, these methods are still impaired by the limited stability of the bioconjugate products and/or by the lack of chemoselectivity of the derivatization process.

**Scheme 17:**
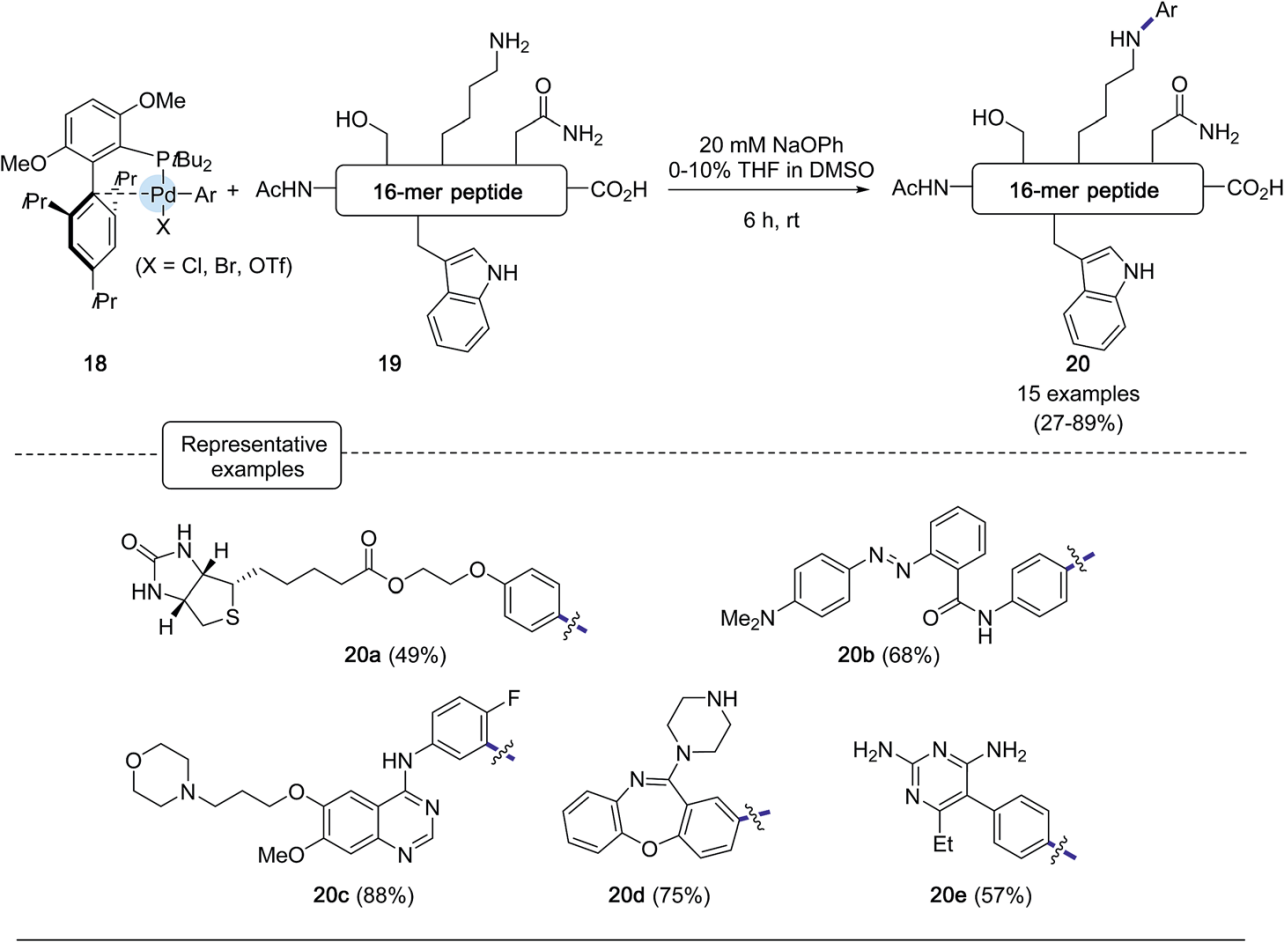
Lysine arylation experiments of AcNH-LSQETFSDLWKLLPEN-CO_2_H.

**Scheme 18:**
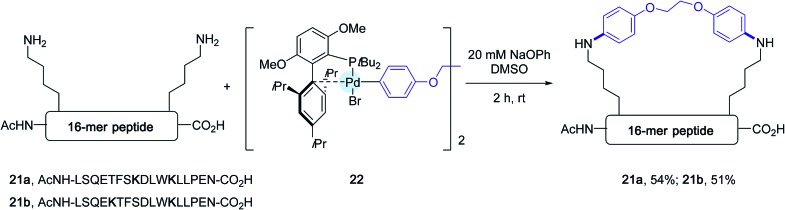
Stapling experiments of 16-mer peptides **21a** and **21b** containing lysine residues at [*i*, *i* + 4] and [*i*, *i* + 7] positions.

In this context, the Buchwald group envisioned to affix aryl appendages to lysine residues with the aid of arylpalladium reagents.^28^ Considering the lower nucleophilicity of lysine ε-amino groups compared to the sulfhydryl group, the authors focused their attention at defining the nature, not only of the ligand to be attached to palladium but also of the base to be added to the reaction medium. Special care was given to the choice of the base, which on the one hand had to be relatively mild to avoid any degradation of the peptide substrates and, on the other hand, strong enough to convert palladium–amine complexes, resulting from the initial attachment of lysine residues, into palladium-amide equivalents.^51^,^52^ As for their C–S bond forging bioconjugation chemistry, the authors privileged ligands from the biaryl phosphine series which have been much in use in Pd-catalyzed amination reactions.^44^ Among them, they eventually highlighted ligands reknown to speed up reductive elimination steps from Pd-amido complexes.^53^ After extensive scouting investigations performed on a model nonapeptide comprising one lysine, the highly sterically hindered *t*BuBrettPhos^44^ (see Scheme 17) and sodium phenoxide, of relatively low basicity, were eventually selected. Using DMSO as the main solvent, an excellent arylation yield of 94% of the lysine residue was obtained after 6 h at room temperature. Importantly, in the presence of the base, no significant signs of decomposition of the peptide substrate were observed. With the optimized conditions in hands, the scope of the arylation protocol was scrutinized on a complex 16-mer peptide as the substrate. As indicated in Scheme 18, the arylation reaction proceeded quite satisfactorily in most cases, yielding to peptide products not only equipped with biotinyl (**20a**) or fluorescent handles (**20b**) but also with moieties derived from complex drug molecules (**20c–20e**).

It should be noted however that the presence of a cysteine residue in the peptide substrates was not tolerated owing to competitive base-mediated dehydroalanine formation. In addition, in the case of peptides comprising a primary amine at the N-terminus or amino acid residues containing an amide or a guanidine side chain, bioconjugation reactions were not chemoselective, giving rise to biarylated products.

In the wake of their macrocyclisation experiments performed on peptide substrates adorned with two cysteines, the authors generated *in situ* the bis-palladium reagent **22** in view of stapling experiments involving two lysine residues. As shown in Scheme 18, again in the presence of sodium phenoxide and in DMSO, two 16-mer peptides **21a** and **21b** containing respectively lysines at [*i*, *i* + 4] and [*i*, *i* + 7] positions could be stapled with comparable efficiencies.

The derivatization of lysine residues with the aid of arylpalladium(ii) reagents has been so far confined to peptide substrates. In spite of its efficiency, extension of the method to proteins is still out of reach, mainly due to the lack of solubility of *t*BuBrettPhos-supported Pd complexes in water.

## V Conclusions

The past seven years witnessed the burgeoning of new protocols for the preparation of protein bioconjugates highlighting the utilization of ready-to-use aryl-palladium and, in one instance, -gold reagents. This novel bioorthogonal chemistry, based on stoichiometric processes, has put in the limelight elaborated complexes equipped with biologically relevant appendages such as fluorescent and affinity tags, as well as drug molecules. Central here has been the choice of the ligands attached to the transition metal in order to confer to aryl complexes, resulting from an oxidative addition step, not only their stability under air and in aqueous environments, but also the desired reactivity toward endogenous nucleophiles as well as functionalities not encountered in Nature. Along this line, this research has already provided new ways for forging C–S and C–C bonds with an excellent chemoselectivity onto proteins, by targeting respectively native cysteine residues or pre-installed terminal alkyne or alkene handles. Whilst, in both cases, experiments have been confined so far to *in vitro* derivatization studies, C–C bond arylation procedures, which involve exogenous residues, may well transition soon to more complex biological settings. The aim would be here to expand the palette of known sustainable alternatives to the gold standard click chemistry that has been quite in vogue in bioconjugation processes during the past decades, in spite of the toxicity issues associated to the utilization of copper species. On the other hand, the C–S bond-forging chemistry should remain more adapted to ‘in-flask’ studies, taking into account the plethora of potentially competing nucleophiles present in the biological milieu. It remains more open to the design of new biotherapeutics, such as antibody-drug conjugates or stapled-peptides and -proteins and should offer, as well, a robust alternative to the venerable maleimide ligation procedure that, in some instances, had suffered from limitations due to the instability of maleimide adducts. In spite of their performance, these novel transition metal mediated reactions still deserve attention in order to compete even more favorably with existing procedures. On the one hand, efforts should be made to disclose, in all cases, co-solvent-free procedures that adhere perfectly to physiological conditions. On the other hand, with the view of adapting arylation reactions to biological environments, kinetics of C–C bond formations should be ameliorated especially in the case of alkene-containing protein substrates. In another context, it would be interesting to extend the strategy to the forging of C–N bonds onto protein substrates. To this end, the derivation procedure of peptide lysine residues just launched by the Buchwald group should be fruitful.^54^

To conclude, although the utilization of transition metal reagents in stoichiometric reactions involving proteins might appear, at first glance, as a ‘to-be-avoided’ option compared to versions based on catalytic processes that have been developing at nearly the same time, the reverse scenario might prevail in the future. Indeed, contrary to bicomponent reactions involving proteins and transition metal reagents only, it is anticipated that the development of catalytic procedures will remain for a long time thwarted by unproductive interactions of metal catalysts with nucleophiles from the biological milieu. An extension of the utilization of aryl transition metal reagents for the derivatization of biomolecules other than proteins^55^ is in addition expected in the near future.

## Conflicts of interest

There are no conflicts to declare.
